# Higher Protein Density Diets Are Associated With Greater Diet Quality and Micronutrient Intake in Healthy Young Adults

**DOI:** 10.3389/fnut.2019.00059

**Published:** 2019-05-07

**Authors:** Jess A. Gwin, J. Philip Karl, Laura J. Lutz, Erin Gaffney-Stomberg, James P. McClung, Stefan M. Pasiakos

**Affiliations:** ^1^Oak Ridge Institute for Science and Education Supporting the Military Nutrition Division of the US Army Research Institute of Environmental Medicine, Natick, MA, United States; ^2^Military Nutrition Division of the US Army Research Institute of Environmental Medicine, Natick, MA, United States; ^3^Military Performance Division of the US Army Research Institute of Environmental Medicine, Natick, MA, United States

**Keywords:** protein, diet quality, micronutrients, shortfall nutrients, healthy eating index

## Abstract

**Objective:** This study characterized habitual dietary protein intake in healthy young adults entering military service and explored whether diet protein density is associated with diet quality and micronutrient intake.

**Methods:** An FFQ was used to estimate habitual dietary intake and calculate HEI scores in 276 males [mean(SD), age:21.1y(3.8)] and 254 females [age:21.2y(3.7)]. Multivariate-adjusted MANCOVA and ANCOVA models were used to identify associations between protein density quartiles and HEI scores and micronutrient intake. Higher HEI components scores for sodium, refined grains, and empty calories indicate lower intake; higher scores for all other components indicate higher intakes.

**Results:** Mean(SD) energy-adjusted protein intakes were 29.3(3.2), 36.0(1.4), 40.8(1.3), and 47.9(3.9) g/1,000 kcal for protein density quartiles 1–4, respectively. For males, empty calorie scores as well as dark green and orange vegetable scores were higher in quartiles 3 and 4 than 1 and 2 (all, *p* < 0.05). Scores for total vegetable, dairy, and total protein foods were lower in quartile 1 vs. quartiles 2, 3, and 4 (all, *p* < 0.05). Sodium scores decreased as quartiles increased (*p* < 0.001). Total HEI, fruit, whole grains, seafood and plant protein, fatty acids, and refined grain scores did not differ. For females, total HEI, vegetable, and total protein foods scores were higher in quartiles 3 and 4 than 1 and 2 (all, *p* < 0.05). Empty calorie scores increased as quartile increased (*p* < 0.05). Dairy scores were higher in quartiles 2, 3, and 4 than 1 (*p* < 0.05). Whole fruit scores were lowest in quartile 1 (*p* < 0.05). Whole grain as well as seafood and plant protein scores were higher in quartile 4 vs. 1 (both, *p* < 0.05). Sodium scores decreased as quartile increased (*p* < 0.001). Fatty acids scores did not differ. For males and females, micronutrient intakes progressively increased across quartiles with the exception of calcium and vitamin C, (all, *p* < 0.05). Intakes remained nearly the same when controlled for fruit and vegetable intake.

**Conclusion:** These cross-sectional data suggest that habitually consuming a higher protein density diet is associated with better scores for some, but not all, diet quality components in males, better overall diet quality scores in females, and greater intakes of micronutrients in both male and female healthy, young adults entering military service.

## Introduction

Dietary protein recommendations are established as the minimum amount of dietary protein intake necessary to maintain nitrogen balance (1). However, accumulating evidence demonstrates that protein intakes above the Recommended Dietary Allowance (RDA; 0.8 g • kg^−1^ • d^−1^ for healthy adults) are metabolically advantageous and may reduce chronic disease risk (2), enhance satiety (3), and body composition during weight loss (4) and exercise training (5). Protein-containing foods are comprised of more than their constituent amino acids; they also contain a high ratio of micronutrients to energy and are therefore nutrient-dense (1). As a result, consuming a higher protein-dense diet, defined as consuming more energy from protein-containing whole foods without increasing total energy intake, may enhance diet quality and improve micronutrient intake (1, 6).

Diet quality is considered a primary modifiable risk factor associated with preventable health complications and chronic disease development (7, 8). Ensuring dietary micronutrient intakes meet minimum requirements is one strategy to optimize diet quality. Optimizing diet quality is particularly important for populations whose health and physical performance are critical to occupational success and resilience to injury and stressors associated with unaccustomed physical training, including healthy young adults entering initial military training (9–11). The Dietary Guidelines for Americans 2015–2020 recently identified several micronutrients that are often underconsumed and are therefore considered shortfall nutrients (12). These include potassium, choline, magnesium, calcium, vitamins A, D, E, and C (12), which serve critical roles in bone health, blood pressure regulation, cancer, and cardiovascular disease prevention (13–16). Suboptimal intakes of iron, folate, zinc, and vitamins B1, B2, B3, and B12 may diminish physical performance and limit beneficial adaptations to physical training (5, 17, 18). Nutrient-dense, protein-containing whole-foods are excellent sources of the aforementioned micronutrients (19, 20). It is also possible that increasing the protein density of the diet may be related to better overall food choices that contribute to better diet quality and micronutrient intake. In contrast it is conceivable that consuming more protein-containing foods may negatively impact diet quality if these foods are higher in saturated-fat and sodium (i.e., processed and non-lean meats). However, whether consuming higher amounts of total energy as protein-containing foods improves or diminishes diet quality and micronutrient intakes is not well-described (1).

This cross-sectional study characterized habitual dietary protein intake in healthy young adults entering military service and explored whether the protein density of the diet was associated with diet quality, as indicated by Healthy Eating Index (HEI) scores and micronutrient intake. We hypothesized that when controlling for energy density and total energy intake, diet quality, and micronutrient intakes would be greater in those consuming higher quantities of dietary protein.

## Methods

This research was carried out in accordance with US Army Regulation 70–25 and the provisions of Title 32 Code of Federal Regulations Part 219 Protection of Human Subjects. This research was approved by the Institutional Review Board at the US Army Research Institute of Environmental Medicine.

### Participants

The study sample included 890 healthy adults (ages 17–42 y) entering initial military training. Data collection occurred over four study iterations which took place as follows: February, 2010 at Fort Jackson, SC (*n* = 223); June 2012 and February 2013 at Fort Sill, OK (*n* = 492); and April 2015 at Fort Jackson, SC (*n* = 175). All data were collected as part of primary studies designed to assess the effects of calcium and vitamin D supplementation on bone health (11, 21, 22). All participants provided informed, written consent.

### Dietary Intake

The 3-month 2005 Block Food Frequency Questionnaire (FFQ) was used to assess dietary intake prior to initial military training accession. This semi-quantitative FFQ captures usual intakes of food groups and nutrients for 3 months prior to administration using a food item list (23). Respondents select the frequency (i.e., never to every day) and quantity of foods they consumed from the food item list, and are asked to specify if foods were modified or standard items (i.e., low-fat vs. full-fat foods). The FFQ is commonly used to assess dietary intakes and is validated for use in the general US population and has been used to assess dietary intake in military populations (24–27). Charts of photographed foods denoting portion sizes were provided to assist in portion size estimation, and registered Dietitians were available to answer participant questions regarding the FFQ. Questionnaires were analyzed by Nutrition Quest (Berkeley, CA, USA) using the US Department of Agriculture Food and Nutrient Database for Studies version 1.0. Analysis included computation macro- and micronutrient intakes in addition to HEI total and component scores. Participants were excluded from analyses if they had missing data or indicated implausible energy intakes (males < 800 or > 5,000 kcal/d; females < 300 or > 4,500 kcal/d) (8, 9). Those that reported consuming supplements at least once per week were also excluded. Demographic information was collected through self-report. Dietary intakes from 276 males [mean (SD), age: 21.1 y (3.8), body mass index: 25.8 kg/m^2^ (3.7)] and 254 females [age: 21.2 y (3.7), BMI: 24.2 kg/m^2^ (2.9)] were included in the final statistical analyses.

### Healthy Eating Index Components

HEI is a diet quality measure that reflects conformance to the Dietary Guidelines for Americans (28). HEI total scores are the composite of 12 component scores and range from 0 to 100; with 100 denoting perfect compliance with the Dietary Guidelines for Americans. HEI component scores are categorized as indicators of adequacy and moderation. Adequacy components included: total fruit, whole fruit, total vegetables, dark green and orange vegetables and legumes (DGOVL), dairy, total protein foods, seafood and plant protein, whole grains, and fatty acids. Higher adequacy component scores indicate higher consumption. Moderation components included: sodium, refined grains, and empty calories (i.e., calories from solid fats, alcohol, and added sugars). Higher moderation component scores indicate lower consumption of these categories. Collectively, higher total and component scores suggest better dietary quality vs. lower scores. HEI 2010 was used for the current analyses to maintain consistency within the data set since a majority of the data had been analyzed by Nutrition Quest (Berkeley, CA, USA) prior to the release of HEI 2015.

### Statistical Analyses

A multivariate-adjusted ANCOVA model was used to identify associations between quartiles of protein intake and means of energy adjusted protein intake as well as means of relative protein intake. A multivariate-adjusted ANCOVA model was used to identify associations between quartiles of protein intake and total HEI score. Multivariate-adjusted MANCOVA models were used to identify associations between quartiles of protein intake and HEI component scores (i.e., total fruit, whole fruit, total vegetables, dark green and orange vegetables and legumes (DGOVL), dairy, total protein foods, seafood and plant protein, whole grains, fatty acids, sodium, refined grains, and empty calories) as well as micronutrients of interest. A sex-by-protein density quartile interaction was detected for the Total HEI and HEI component scores. Therefore, these data was analyzed for each sex separately (i.e., Model 1). Model 1 was adjusted for study iteration, age, ethnicity, race, physical activity, smoking status, energy density (kcal/g of food consumed) and total energy intake. No sex-by-protein density quartile interaction was detected for micronutrient intakes. Thus, sex was added to Model 2 as a covariate. For Model 3, fruit and vegetable intakes were added to Model 2 as covariates. Race was categorized as white, black, or other. Ethnicity was categorized as Hispanic or non-Hispanic. Habitual physical activity was categorized as yes (i.e., at least one time per week) or no (i.e., never or rarely). Smoking habits were categorized as yes (i.e., current smoker) or no (i.e., non-smoker or former smoker). Data were analyzed using the Statistical Package for the Social Sciences (version 24.0; IBM SPSS). A Bonferroni correction was applied to correct for multiple comparisons. All results are presented as mean (SD) as appropriate. Adherence to model assumptions was verified and statistical significance was set at *p* < 0.05.

## Results

### Protein Density Quartiles

Mean ± SD energy-adjusted protein intakes increased across protein density quartiles [29.3 (3.2), 36.1 (1.4), 40.9 (1.3), and 47.9 (3.9) g/1,000 kcal, respectively; all, *p* < 0.05; Table 1)]. Protein intakes expressed as percent of total energy intake increased across protein density quartiles (11.7 (1.3), 14.4 (0.6), 16.4 (0.5), and 19.2 (1.6) percent, respectively; all, *p* < 0.05). Relative protein intakes across protein density quartiles were lower in quartile 1 [0.9 (0.5)] than quartiles 2, 3, and 4 (1.2 (0.6), 1.3 (0.6), and 1.3 (0.5) g ∙ kg^•1^ ∙ d^•1^, respectively; all, *p* < 0.05.

**Table 1:** Habitual estimated protein intake by protein density quartile in healthy young adults^1^.

**Protein intake**	**Protein density quartile**			
	**1 (*n* = 135)**	**2 (*n* = 134)**	**3 (*n* = 134)**	**4 (*n* = 127)**
g/1,000 kcal^2^	29.3 ± 3.2^a^	36.1 ± 1.4^b^	40.9 ± 1.3^c^	47.9 ± 3.9^d^
Percent total energy^3^	11.7 ± 1.3^a^	14.4 ± 0.6^b^	16.4 ± 0.5^c^	19.2 ± 1.6^d^
g/kg body weight^3^	0.9 ± 0.5^a^	1.2 ± 0.6^b^	1.3 ± 0.6^b^	1.3 ± 0.5^b^

1 Values are means (SD).

2 Data are analyzed using ANCOVA adjusted for age, ethnicity, race, physical activity, smoking status, and total energy intake, within a row, values not sharing superscript letters are different at the univariate level, p < 0.05.

3 *Data are analyzed using ANCOVA adjusted for age, ethnicity, race, physical activity, smoking status, and energy density within a row, values not sharing superscript letters are different at the univariate level, p < 0.05*.

### HEI Total and Component Scores

For males empty calorie and DGOVL component scores were higher in protein density quartiles 3 and 4 than 1 and 2 (all, *p* < 0.05; Table 2). Component scores for total vegetable, dairy, and total protein food consumption were lower in protein density quartile 1 compared to quartiles 2, 3, and 4 (all, *p* < 0.05). Sodium component scores decreased progressively as protein density quartile increased (*p* < 0.001). Total HEI, total fruit, whole fruit, whole grains, seafood and plant protein, fatty acids, and refined grain scores did not differ across protein density quartiles.

**Table 2:** Healthy Eating Index (HEI) scores based on habitual protein intake in healthy young males^1^^,^ ^2^.

**HEI score**	**Protein density quartile**				***P*-value^4^**
	**1 (*n* = 54)**	**2 (*n* = 70)**	**3 (*n* = 78)**	**4 (*n* = 74)**	
Total HEI score^3^	51.3 ± 12.1	53.5 ± 12.6	54.7 ± 10.2	56.1 ± 9.7	*P* = 0.22
Total fruit	3.3 ± 1.6	3.2 ± 1.6	2.8 ± 1.5	2.8 ± 1.4	*P* = 0.03
Whole fruit	2.9 ± 1.5	2.9 ± 1.7	2.6 ± 1.7	2.9 ± 1.6	*P* = 0.85
Total vegetables	2.7 ± 1.0^a^	3.0 ± 1.2^a, b^	3.3 ± 1.1^b^	3.5 ± 1.2^b^	*P* = 0.003
DGOVL^5^	1.9 ± 1.8^a^	2.5 ± 1.8^a^	3.3 ± 1.7^b^	3.3 ± 1.7^b^	*P* = 0.001
Whole grains	2.2 ± 1.8	2.8 ± 2.3	3.2 ± 2.5	3.2 ± 2.6	*P* = 0.12
Dairy	4.8 ± 2.1^a^	6.1 ± 2.3^b^	6.3 ± 2.4^b^	5.7 ± 2.7^b^	*P* = 0.001
Total protein foods	4.2 ± 0.8^a^	4.7 ± 0.5^b^	4.9 ± 0.3^b, c^	5.0 ± 0.3^c^	*P* = 0.001
Seafood and plant protein	3.1 ± 1.5	3.2 ± 1.5	3.0 ± 1.6	3.4 ± 1.6	*P* = 0.45
Fatty acids	5.1 ± 2.8	4.3 ± 2.6	4.7 ± 2.2	4.5 ± 2.3	*P* = 0.14
Sodium^6^	6.3 ± 1.7^a^	4.6 ± 1.9^b^	3.1 ± 1.9^c^	2.2 ± 1.8^d^	*P* = 0.001
Refined grains^6^	8.0 ± 2.4	7.6 ± 2.2	7.1 ± 2.3	7.8 ± 2.1	*P* = 0.08
Empty calories^6^	6.9 ± 5.9^a^	8.4 ± 5.1^a^	10.5 ± 3.7^b^	11.9 ± 3.6^b^	*P* = 0.001

1 Values are mean ± SD.

2 Data are analyzed using MANCOVA are adjusted for age, ethnicity, race, physical activity, smoking status, energy density, and total energy intake, within a row, values not sharing superscript letters are different at the univariate level, p < 0.05.

3 Data are analyzed using ANCOVA adjusted for age, ethnicity, race, physical activity, smoking status, energy density, and total energy intake, within a row, values not sharing superscript letters are different at the univariate level, p < 0.05.

4 P-value < 0.05 indicates a main effect of protein density quartile.

5 Dark green and orange vegetables and legumes (DGOVL).

6 *Moderation components higher scores reflect lower intakes*.

For females total HEI, total vegetables, DGOVL, and total protein foods were higher in protein density quartiles 3 and 4 than quartiles 1 and 2 (all, *p* < 0.05; Table 3). Empty calorie component scores increased as protein density quartile increased (*p* < 0.05). Dairy component scores were higher in quartiles 2, 3, and 4 than quartile 1 (*p* < 0.05). Refined grain component scores were lowest in protein density quartile 2 and whole fruit scores were lowest in quartile 1 (both, *p* < 0.05). Seafood and plant protein as well as whole grain consumption were only higher in protein density quartile 4 compared to quartile 1 (both, *p* < 0.05). Sodium component scores decreased progressively as protein density quartile increased (*p* < 0.001). Fatty acids component scores did not differ across protein density quartiles.

**Table 3:** Healthy Eating Index (HEI) scores based on habitual protein intake in healthy young females^1^^,^ ^2^.

**HEI score**	**Protein density quartile**				***P*-value^4^**
	**1 (*n* = 81)**	**2 (*n* = 64)**	**3 (*n* = 56)**	**4 (*n* = 53)**	
Total HEI score^3^	51.7 ± 11.2^a^	54.1 ± 10.9^a^	61.7 ± 11.9^b^	66.4 ± 12.3^b^	*P* = 0.001
Total fruit	3.1 ± 1.7	3.4 ± 1.7	3.8 ± 1.4	3.9 ± 1.4	*P* = 0.05
Whole fruit	2.9 ± 1.7^a^	3.1 ± 1.6^a, b^	3.8 ± 1.3^b^	4.0 ± 1.4^b^	*P* = 0.001
Total vegetables	2.8 ± 1.3^a^	3.0 ± 1.2^a^	3.8 ± 1.1^b^	4.1 ± 1.1^b^	*P* = 0.001
DGOVL^5^	2.3 ± 1.8^a^	2.6 ± 1.7^a^	4.0 ± 1.5^b^	4.3 ± 1.3^b^	*P* = 0.001
Whole grains	2.4 ± 1.9^a^	3.0 ± 2.4^a, b^	3.1 ± 2.6^a, b^	3.6 ± 2.6^b^	*P* = 0.03
Dairy	3.9 ± 1.9^a^	6.2 ± 2.5^b^	6.2 ± 2.8^b^	7.0 ± 2.8^b^	*P* = 0.001
Total protein foods	3.9 ± 0.9^a^	4.4 ± 0.8^b^	4.8 ± 0.5^c^	5.0 ± 0.2^c^	*P* = 0.001
Seafood and plant protein	2.9 ± 1.5^a^	3.2 ± 1.5^a, b^	3.6 ± 1.5^a, b^	3.9 ± 1.4^b^	*P* = 0.006
Fatty acids	5.5 ± 2.9	4.5 ± 2.6	5.4 ± 2.9	5.6 ± 2.8	*P* = 0.13
Sodium^6^	6.6 ± 2.5^a^	4.3 ± 2.0^b^	3.5 ± 2.1^b, c^	2.5 ± 2.0^c^	*P* = 0.001
Refined grains^6^	8.0 ± 2.6^a, b^	6.8 ± 2.9^a^	8.3 ± 2.2^b^	8.5 ± 2.1^b^	*P* = 0.01
Empty calories^6^	7.2 ± 5.4^a^	9.5 ± 3.9^b^	11.5 ± 4.1^c^	14.0 ± 4.0^d^	*P* = 0.001

1 Values are mean ± SD.

2 Data are analyzed using MANCOVA are adjusted for age, ethnicity, race, physical activity, smoking status, energy density, and total energy intake, within a row, values not sharing superscript letters are different at the univariate level, p < 0.05.

3 Data are analyzed using ANCOVA adjusted for age, ethnicity, race, physical activity, smoking status, energy density, and total energy intake, within a row, values not sharing superscript letters are different at the univariate level, p < 0.05.

4 P-value < 0.05 indicates a main effect of protein density quartile.

5 Dark green and orange vegetables and legumes (DGOVL).

6 *Moderation components higher scores reflect lower intakes*.

### Micronutrient Intakes

Composite micronutrient intake differed (*p* < 0.001) by protein density quartile in both model 2 and 3. For model 2, individual micronutrient intakes, except calcium and vitamin C, progressively increased (all, *p* < 0.05) with increasing protein density quartiles (Table 4). Calcium intakes in protein density quartile 1 were lower than quartiles 2, 3, and 4 (*p* < 0.001). Vitamin C intakes were not different across protein density quartiles. Model 3 indicated that independent of fruit and vegetable intake, all micronutrients progressively increased across protein density quartiles except vitamins A, E, C, and folate (all, *p* < 0.05) (Table 5). Vitamin A and C intakes in protein density quartile 1 were lower than quartiles 2, 3, and 4 (all, *p* < 0.001). Vitamin E intakes were not different across protein density quartiles. There was not a protein density quartile-by-sex interaction indicated by model 3, (*p* = 0.13).

**Table 4:** Estimated daily micronutrient intakes across protein density quartile in healthy young adults^1^^,^ ^2^.

**Micronutrients of interest**	**Protein density quartile**				***P*-value^3^**
	**1 (*n* = 135)**	**2 (*n* = 134)**	**3 (*n* = 134)**	**4 (*n* = 127)**	
Potassium (mg)	1092.0 ± 316.7^a^	1207.6 ± 291.9^b^	1304.2 ± 292.9^c^	1442.7 ± 334.8^d^	*P* = 0.001
Calcium (mg)	356.6 ± 110.1^a^	438.1 ± 114.0^b^	449.2 ± 123.8^b^	482.8 ± 178.2^b^	*P* = 0.001
Magnesium (mg)	117.8 ± 27.4^a^	134.2 ± 32.5^b^	150.1 ± 39.6^c^	163.0 ± 41.5^c^	*P* = 0.001
Folate (mg)	101.0 ± 45.6^a^	112.9 ± 47.6^a^	132.5 ± 54.4^b^	148.1 ± 73.7^b^	*P* = 0.001
Choline (mg)	123.5 ± 30.0^a^	150.0 ± 34.0^b^	173.9 ± 39.5^c^	205.6 ± 58.1^d^	*P* = 0.001
Iron (mg)	6.1 ± 1.5^a^	6.8 ± 1.3^b^	7.3 ± 1.7^b^	8.0 ± 2.0^c^	*P* = 0.001
Zinc (mg)	4.5 ± 1.0^a^	5.4 ± 0.7^b^	6.2 ± 1.2^c^	7.2 ± 2.1^d^	*P* = 0.001
Vitamin A (retinol activity eq., mcg)	283.2 ± 118.0^a^	346.2 ± 147.0^b^	419.0 ± 245.6^c^	446.1 ± 216.4^c^	*P* = 0.001
Vitamin C (mg)	68.1 ± 44.5	58.5 ± 34.6	58.1 ± 31.3	62.3 ± 34.3	*P* = 0.56
Vitamin D (IU)	50.0 ± 37.1^a^	73.7 ± 43.7^b^	84.7 ± 61.1^b^	103.3 ± 58.6^c^	*P* = 0.001
Vitamin E (alpha-tocopherol, mg)	3.5 ± 1.1^a^	3.4 ± 1.0^a, b^	3.8 ± 1.2^a^	3.9 ± 1.5^a, c^	*P* = 0.001
Vitamin B1 (mg)	0.7 ± 0.2^a^	0.8 ± 0.2^b^	0.8 ± 0.2^b^	0.9 ± 0.2^c^	*P* = 0.001
Vitamin B2 (mg)	0.8 ± 0.2^a^	1.0 ± 0.3^b^	1.1 ± 0.3^b^	1.2 ± 0.3^c^	*P* = 0.001
Vitamin B3 (mg)	8.8 ± 2.1^a^	10.3 ± 2.1^b^	11.2 ± 2.3^c^	13.1 ± 2.8^d^	*P* = 0.001
Vitamin B12 (mcg)	1.9 ± 0.7^a^	2.5 ± 0.6^b^	2.9 ± 1.0^c^	3.7 ± 1.2^d^	*P* = 0.001

1 Values are mean ± SD.

2 Data are analyzed using MANCOVA adjusted for age, sex, ethnicity, race, physical activity, smoking status, energy density, and total energy intake, within a row, values not sharing superscript letters are different at the univariate level, p < 0.05.

3 *P-value < 0.05 indicates a main effect of protein density quartile*.

**Table 5:** Estimated daily micronutrient intakes across protein density quartile when adjusted for fruit and vegetable intake in healthy young adults^1^^,^ ^2^.

**Micronutrients of interest**	**Protein density quartile**				***P*-value^3^**
	**1 (*n* = 135)**	**2 (*n* = 134)**	**3 (*n* = 134)**	**4 (*n* = 127)**	
Potassium (mg)	1092.0 ± 316.7^a^	1207.6 ± 291.9^b^	1304.2 ± 292.9^b^	1442.7 ± 334.8^c^	*P* = 0.001
Calcium (mg)	356.6 ± 110.1^a^	438.1 ± 114.0^b^	449.2 ± 123.8^b, c^	482.8 ± 178.2^c^	*P* = 0.001
Magnesium (mg)	117.8 ± 27.4^a^	134.2 ± 32.5^b^	150.1 ± 39.6^b^	163.0 ± 41.5^c^	*P* = 0.001
Folate (mg)	101.0 ± 45.6^a^	112.9 ± 47.6^a, b^	132.5 ± 54.4^a, b^	148.1 ± 73.7^b^	*P* = 0.001
Choline (mg)	123.5 ± 30.0^a^	150.0 ± 34.0^b^	173.9 ± 39.5^c^	205.6 ± 58.1^d^	*P* = 0.001
Iron (mg)	6.1 ± 1.5^a^	6.8 ± 1.3^b^	7.3 ± 1.7^b^	8.0 ± 2.0^c^	*P* = 0.001
Zinc (mg)	4.5 ± 1.0^a^	5.4 ± 0.7^b^	6.2 ± 1.2^c^	7.2 ± 2.1^d^	*P* = 0.001
Vitamin A (retinol activity eq., mcg)	283.2 ± 118.0^a^	346.2 ± 147.0^b^	419.0 ± 245.6^b^	446.1 ± 216.4^b^	*P* = 0.001
Vitamin C (mg)	68.1 ± 44.5^a^	58.5 ± 34.6^b^	58.1 ± 31.3^b^	62.3 ± 34.3^b^	*P* = 0.02
Vitamin D (IU)	50.0 ± 37.1^a^	73.7 ± 43.7^b^	84.7 ± 61.1^c^	103.3 ± 58.6^d^	*P* = 0.001
Vitamin E (alpha-tocopherol, mg)	3.5 ± 1.1	3.4 ± 1.0	3.8 ± 1.2	3.9 ± 1.5	*P* = 0.25
Vitamin B1 (mg)	0.7 ± 0.2^a^	0.8 ± 0.2^b^	0.8 ± 0.2^b^	0.9 ± 0.2^c^	*P* = 0.001
Vitamin B2 (mg)	0.8 ± 0.2^a^	1.0 ± 0.3^b^	1.1 ± 0.3^b^	1.2 ± 0.3^c^	*P* = 0.001
Vitamin B3 (mg)	8.8 ± 2.1^a^	10.3 ± 2.1^b^	11.2 ± 2.3^b^	13.1 ± 2.8^c^	*P* = 0.001
Vitamin B12 (mcg)	1.9 ± 0.7^a^	2.5 ± 0.6^b^	2.9 ± 1.0^c^	3.7 ± 1.2^d^	*P* = 0.001

1 Values are means (SD).

2 Data are analyzed using MANCOVA adjusted for age, sex, ethnicity, race, physical activity, smoking status, energy density, total energy intake, fruit intake, and vegetable intake, within a row, values not sharing superscript letters are different at the univariate level, p < 0.05.

3 *P-value < 0.05 indicates a main effect of protein density quartile*.

## Discussion

This cross-sectional study assessed whether consuming greater amounts of dietary protein, resulting in higher diet protein density, was associated with diet quality and dietary micronutrient intakes in healthy young adults. We demonstrate that habitually consuming high protein density diets, independent of energy density and total energy intake, was associated with better scores for select diet quality components in males, better overall diet quality scores in females, and greater micronutrient intakes for both males and females. We also demonstrate that the relationship between protein density and intake of several micronutrients was independent of fruit and vegetable intake. These associative data suggest that consuming a high proportion of total energy derived from protein-containing whole foods supports healthy dietary intake patterns that align with current nutrition guidelines for Americans.

To our knowledge, only one other study (29) has addressed whether consuming greater amounts of protein-containing foods was associated with diet quality in healthy adults. In that study total protein intake was negatively associated with diet quality in males but positively associated with diet quality in females. In addition, animal-based protein intake was negatively associated with diet quality in males, whereas animal-based protein intake was positively associated with diet quality in females. Regardless of sex, plant-based protein intake was positively associated with better diet quality. Similarly, we demonstrate that consuming a higher protein-dense diet was associated with consuming more protein from seafood and plants than lower protein-dense diets, but only in females. It is possible that, in this population, males may consume more of their protein from animal sources, whereas in females there may be a larger contribution from plant-based sources. The remaining divergent associations between diet quality, protein quantity, and intakes of animal- and plant-based protein foods (i.e., without including seafood proteins) in the Camilleri study are difficult to reconcile with our findings, which may be largely a function of the diet scoring methodologies used. HEI estimates dietary conformance to the Dietary Guidelines for Americans, whereas the PANDiet index used by Camilleri et al. estimates the probability that usual dietary intakes meet French and or European Union nutritional recommendations (28, 29). Nevertheless, the data from Camilleri et al. highlight an important analytical limitation of the HEI, which does not provide specific examination of all protein food sources or the characteristics of these food items (i.e., lean meats or low-fat dairy). As such, the potential for animal- and plant-based protein foods to be differentially related to diet quality in healthy young adults entering military service cannot be discerned. We were also unable to identify which food sources were responsible for the increasing sodium intakes across protein density quartile. It is possible that the individuals in the highest protein density quartile consumed more processed protein-containing foods, which are typically higher in sodium (30). Lastly, at the time of data analysis, the HEI 2010 was the current method for assessing diet quality and conformance to the Dietary Guidelines for Americans. Future analyses in similar cross-sectional studies using the HEI 2015 would allow for differentiation of added sugars and saturated fats that is unattainable within the empty calories component score of the HEI 2010.

The Dietary Guidelines for Americans recommend a shift toward consuming nutrient-dense foods at the expense of limiting empty calorie intake (i.e., energy from solid fats, added sugars, and alcohol) (12). In the current study, we demonstrate that those in the highest dietary protein density quartile also habitually consumed more total vegetables, including nutrient-dense dark greens, orange vegetables, and legumes, more whole grains (females only), more dairy, and less empty calories. The apparent protein-related increases in dietary nutrient-density and diet quality were not a function of simply consuming more food (i.e., total energy), but rather a combination of consuming more nutrient-dense foods, and fewer nutrient-poor foods that contain empty calories. These findings are comparable to those derived from NHANES 2003–2004, which suggest that those consuming greater amounts of nutrient-dense foods, not protein *per se*, limited empty calorie intake (31). These findings may be particularly beneficial in the context of healthy weight management and are corroborated by data from other prospective, cross-sectional studies demonstrating lower central adiposity and body mass index in American adults habitually consuming more protein, independent of total energy intake (32, 33).

Individuals in the highest dietary protein density quartile also consumed more non-protein, nutrient-rich foods. This could suggest that the relationship between protein density of the diet and diet quality was mediated by those non-protein foods and general eating habits rather than solely the protein density of the diet. However, when controlling for fruit and vegetable intake, micronutrient intakes across protein density quartiles remained nearly the same. This suggests that food sources of protein within a protein-dense diet are related to nutrient intakes. However, we did not examine whether individuals who consumed a diet with a higher protein density were doing so as part of a strategy to eat an overall healthier diet. Thus, future prospective studies are required to determine causation and identify which food sources of protein may drive this relationship. Additionally, relative protein intakes in the current study were above the RDA, but within the Acceptable Macronutrient Distribution Range for protein (10–35% of total calories) (34). These findings align with our previous observations that protein intakes are generally higher than the RDA in similar military populations (35) and free-living Americans (36).

The potential impact of inadequate micronutrient intake cannot be delineated in the current study, although the biological functions of micronutrients suggest suboptimal intakes may hinder physiologic adaptations and performance during strenuous, unaccustomed physical training (9). Specifically, suboptimal intakes of vitamin D and iron have been recognized to have detrimental effects on health and performance in those entering the military (9–11, 37). Intakes of these nutrients progressively increased as protein density quartile increased suggesting consuming more protein may be beneficial. While the effects of folate and vitamin E on performance are not well-studied, in general suboptimal intakes of folate and vitamin E raise concern as folate is vital for cellular synthesis, growth, and repair (38) and vitamin E is a key antioxidant and contributes to anti-inflammatory processes (39). Similarly, low magnesium intake would suggest a potential greater risk of inefficient energy metabolism and suboptimal neuro-muscular function (40). Although it would be helpful to understand how the estimated micronutrient intakes across protein density quartiles compares to recommended intakes, we cannot directly compare micronutrient intake adequacies to the DRIs due to limitations of the FFQ (41, 42). For example, FFQs rely on single time-point data collection to estimate food intake. Multiple days of direct dietary intake assessment are required when determining adequacy of nutrient intakes (41, 42). However, these data do suggest that increasing the protein density of the diet does seem to relate to better overall diet quality, in this population of healthy young people.

While we were not able to directly address the effects of dietary protein on muscle and performance in the current study, it is reasonable to speculate that higher quality, higher protein diet patterns positively influence skeletal muscle mass, adaptations to exercise, and physical performance. Higher protein intakes offset protein catabolism and support nitrogen balance in individuals exposed to aerobic exercise training (43, 44). Dietary protein, and its constituent amino acids, are also a primary determinant of skeletal muscle protein turnover. Thus, dietary patterns that support routine high-quality protein ingestion, particularly following exercise, should promote beneficial adaptations to training, and facilitate repair and remodeling of existing muscle protein, and accretion of new muscle protein mass (45). The well-established effects of dietary protein on muscle integrity would support shifting dietary patterns in favor of protein dense foods and such a shift would not reduce diet quality since consuming a higher protein density diet appears possible without displacing other nutrient rich, non-protein foods that contribute an overall healthy diet.

## Conclusion

This study demonstrated that habitually consuming more protein resulting in a diet with a higher protein density is associated with better scores for some, but not all, diet quality components in males, better overall diet quality scores in females, and greater intakes of micronutrients in both young male and female adults prior to reporting for military service.

## Data Availability

The ethics approval given by the Institutional Review Board at the US Army Research Institute of Environmental Medicine was given on the provision that the data would not be shared with researchers upon request.

## Ethics Statement

This research was approved by the Institutional Review Board at the US Army Research Institute of Environmental Medicine. Investigators adhered to US Army Regulation 70-25 and the research was conducted in adherence with the provisions of Title 32 Code of Federal Regulations Part 219 Protection of Human Subjects. The consent procedure used was an informed, written consent.

## Disclosure

The views and assertions expressed herein are those of the authors and do not reflect the official policy of the Department of Army, Department of Defense, or the US Government. Any citations of commercial organizations and trade names in this report do not constitute an official Department of the Army endorsement of approval of the products or services of these organizations.

## Author Contributions

JG and SP had primary responsibility for the final content and wrote the manuscript. JG and JK analyzed the data. JG, PK, LL, EG-S, JM, and SP designed the research and approved the final paper.

### Conflict of Interest Statement

The authors declare that the research was conducted in the absence of any commercial or financial relationships that could be construed as a potential conflict of interest.

## Abbreviations

DGOVL: Dark green and orange vegetables and legumes
FFQ: food frequency questionnaire
HEI: Healthy Eating Index
RDA: recommended dietary allowance.
